# Flexible Supercapacitor Electrodes Based on Carbon Cloth-Supported LaMnO_3_/MnO Nano-Arrays by One-Step Electrodeposition

**DOI:** 10.3390/nano9121676

**Published:** 2019-11-24

**Authors:** Pianpian Ma, Na Lei, Bo Yu, Yongkun Liu, Guohua Jiang, Jianming Dai, Shuhong Li, Qiuling Lu

**Affiliations:** 1 School of Materials Science and Engineering, Zhejiang Sci-Tech University, Hangzhou 310018, China; A17857121453@126.com (N.L.); eric_yubo@126.com (B.Y.);; 2 Key Laboratory of ATMMT Ministry of Education & National Engineering Laboratory for Textile Fiber Materials and Processing Technology & Institute of Smart Fiber Materials, Zhejiang Sci-Tech University, Hangzhou 310018, China

**Keywords:** perovskite-type LaMnO_3_/MnO, supercapacitor flexible electrode, electrochemical properties, carbon cloth, electrodeposition

## Abstract

La-based perovskite-type oxide is a new type of supercapacitor electrode material with great potential. In the present study, LaMnO_3_/MnO (LMO/MnO) nano-arrays supported by carbon cloth are prepared via a simple one-step electrodeposition as flexible supercapacitor electrodes. The structure, deposit morphology of LMO/MnO, and the corresponding electrochemical properties have been investigated in detail. Carbon cloth-supported LMO/MnO electrode exhibits a specific capacitance of 260 F·g^−1^ at a current density of 0.5 A·g^−1^ in 0.5 M Na_2_SO_4_ aqueous electrolyte solution. The cooperative effects of LMO and MnO, as well as the uniform nano-array morphology contribute to the good electrochemical performance. In addition, a symmetric supercapacitor with a wide voltage window of 2 V is fabricated, showing a high energy density of 28.15 Wh·kg^−1^ at a power density of 745 W·kg^−1^. The specific capacitance drops to 65% retention after the first 500 cycles due to the element leaching effect and partial flaking of LMO/MnO, yet remains stable until 5000 cycles. It is the first time that La-based perovskite has been exploited for flexible supercapacitor applications, and further optimization is expected.

## 1. Introduction

In recent years, new energy storage devices represented by supercapacitors and lithium-ion batteries have gained significant attention for the sustainable development of resources and environment [1,2,3]. Supercapacitors are identified as the bridge between lithium-ion batteries and conventional dielectric capacitors [4]. They exhibit higher power density and longer cycle life than lithium-ion batteries, while possessing higher energy density and smaller size by comparison to traditional dielectric capacitors, leading to the gradual emergence in many high-tech fields as promising energy storage devices [5,6].

Actually, the performance of the electrode material plays an important role to determine the energy storage characteristics of supercapacitors. Carbon-based materials, conducting polymers, and transition metal oxides/sulfides have been extensively researched as electrode materials for supercapacitor [2,7]. Among them, transition metal oxides/sulfides have attracted public attention on account of their higher special capacitance compared with carbon-based materials as well as preferable cycling stability compared with conducting polymers. However, the application of transition metal oxides/sulfides (MnO_2_, Fe_2_O_3_, ZnO, Ni-Co-S, etc.) is seriously restricted by low electrical conductivity, limited cycling stability, or narrow potential windows [8,9,10,11]. Consequently, it is significant to develop a new type of material that can intrinsically optimize the electrochemical parameters.

La-based perovskite oxides La*B*O_3_ (*B* = Mn, Ni, Co, etc.) have been regarded as prospective materials for protective coatings in solid oxide fuel cells (SOFCs) owing to their structural and chemical stability at high temperature, as well as the high electrical conductivity [12,13]. The special crystal structure and physicochemical properties also indicate their great potential as supercapacitor materials for fast energy storage applications. In 2014, Mefford et al. [14] put forward the anion-intercalation mechanism of LaMnO_3_ perovskite as electrode material of supercapacitors for the first time, and the oxygen-vacancy tailored redox process is also initially proposed for fast energy storage. Since then, a series of researches on the electrochemical behavior of perovskite oxides have been conducted successively [15,16,17]. As a new kind of promising supercapacitor material, La-based perovskite oxides show numerous advantages, such as improved electrical conductivity, wide voltage window (larger than 1 V), and good cycling stability. For A*B*O_3_ perovskite oxides, the electrical conductivity and the corresponding electrochemical properties depend strongly on the contained oxygen-vacancy, which can be adjusted by partial displacement of A-site and/or B-site cations in perovskite structure.

Powder samples prepared by sol–gel approach are commonly used to investigate the electrochemical properties of perovskite oxides, such as La_0.85_Sr_0.15_MnO_3_ [15,18,19] and La_1−x_Ca_x_MnO_3_ [20]. However, powder samples are prone to agglomerate, reducing the specific surface area of the material, which in turn affects the electrochemical properties. Numerous efforts have been made to prevent the aggregation of perovskite powders, and various morphologies with increased surface area were obtained. An urchin-like La_0.8_Sr_0.2_MnO_3_ perovskite oxide with a high specific surface area of 48 m^2^·g^−1^ has been synthesized via a co-precipitation method [21]. Highly porous LaMnO_3_ particles were prepared with polyvinylpyrrolidone (PVP) as a structure directing agent, showing a specific surface area of 47.13 m^2^·g^−1^ [22]. In addition, a series of perovskite oxides with fiber morphology have been prepared by electrospinning process, displaying great potential as electrode materials of supercapacitors [23,24]. Nevertheless, the methods mentioned above involve multistep processes which are complicated and time-consuming. Moreover, conductors (acetylene black, graphite, etc.) and binders (polyvinylidene fluoride PVDF, polytetrafluoroethylene PTFE) are necessary during the electrode preparation, which surely have a non-negligible influence upon the electrochemical characterization of active materials. Meanwhile, the coating process also makes a difference to the electrochemical properties and cycle stability. Therefore, it should be an important issue to explore new structural morphology of perovskite oxides by alternative methods for energy storage applications.

Nowadays, flexible supercapacitors have gradually approached to people’s attention with the rise of portable energy devices. Generally, one of the most valid ways to prepare flexible electrode is to load the active materials on a flexible conductive support [10,25,26]. Carbon cloth, as a novel conductive support, has drawn a large amount of attention due to the low-cost, chemical inertness, robustness, and large specific surface area. For instance, Ni-Co-S nanosheet arrays have been deposited on flexible carbon cloth, and an exceptional energy storage performance is obtained in the prepared flexible electrode [27]. In fact, electrodeposition is a promising approach to prepare various metal oxides, including perovskite oxides [12,28,29]. Moreover, the physicochemical properties suitable for their specific applications can be tailored by changing the deposition parameters (pH and composition of bath, deposition potential/current, time, temperature, etc.).

Herein, we design carbon cloth-supported LaMnO_3_/MnO nano-arrays by a facile electrodeposition method and develop their application as flexible supercapacitor electrodes. The structure, deposit morphology, and the corresponding electrochemical properties have been systematically investigated. The present work provides a new structural morphology (two-dimensional nanostructure) of perovskite oxides as electrodes for supercapacitors. The use of La-based perovskite for the application of flexible supercapacitors by electrodeposition has rarely been reported before and is of research value and practical significance.

## 2. Materials and Methods 

### 2.1. Materials

Lanthanum nitrate hexahydrate (La(NO_3_)_3_·6H_2_O, 99.9%), 50% manganese nitrate solution (50% Mn(NO_3_)_2_), anhydrous sodium sulfate (Na_2_SO_4_, ≥99.9%), and sodium hydroxide powder (NaOH, ≥99.9%) were purchased from Aladdin Co., Ltd. (Shanghai, China) and used without further purification.

### 2.2. Materials Preparation

The flexible carbon cloth-supported LaMnO_3_/MnO (denoted in the following as LMO/MnO) electrode was prepared by electrodeposition method. Firstly, carbon cloth (CeTech Co. Ltd., Taiwan. WOS1009, about 12 mg·cm^−2^, 1.5 cm × 1.5 cm) was immersed in nitric acid for 12 h before deposition. Then, the carbon cloth was successively ultrasound (KQ-400KDE, Kunshan, China) with ethanol and deionized water for 1 h, respectively, followed by 6 h drying in a vacuum at 60 °C. The processed carbon cloth was used as the working electrode, while Ag/AgCl electrode and platinum electrode were respectively set as the reference electrode and the counter electrode. The electrodeposition bath solution was prepared by dissolving nitrites in 60 mL of deionized water: La(NO_3_)_3_·6H_2_O in 2 M concentration and Mn(NO_3_)_2_ in different concentrations (0.1 M, 0.01 M and 0.005 M) in order to obtain different deposit morphologies. The pH of electrodeposition solution was adjusted to about 6.9 by adding appropriate 0.5 M NaOH. Then, electrodeposition was carried out at 50 °C in a three-electrode system with a constant current of 0.5 mA·cm^−2^. An electrochemical workstation (CHI660E, Shanghai Chenhua, China) was the power supply. The as-obtained sample was washed with deionized water repeatedly, and then was dried for 12 h in a vacuum. Finally, it was annealed at 800 °C for 4 h under N_2_ atmosphere and the mass loading of electrode was about 0.5 mg.

### 2.3. Phase and Structural Characterization

The phase composition and crystal structure of LMO/MnO were identified by an X-ray diffractometer (XRD, Bruker AXS D8-discover, Karlsruhe, Germany), which was conducted directly on the deposited sample. The excitation light source was CuKα radiation with λ = 0.154056 nm, and the scan rate was 2°·min^−1^ at the 2θ range of 10°–90°. Samples were cut into small pieces (3 mm × 3 mm), and stick to a sample stage with conductive adhesive for surface morphology observation, which was performed by field emission scanning electron microscopy (FE-SEM) (ULTRA-55, Zeiss, Oberkochen, Germany). Samples for transmission electron microscopy (TEM) were prepared by dissolving LMO/MnO in acetone and dispersing the suspension onto a holey carbon 200 mesh TEM grid. TEM investigations were performed on 200 kV electron microscopy (Tecnai G2 F20 S-TWIN, FEI Co., Hillsboro, OR, USA) equipped with an X-ray spectrometer (EDAX Analyzer (DPP-П), Edax Inc., Mahwah, NJ, USA) for energy dispersive spectroscopy (EDS) analysis. The surface electronic states were characterized by X-ray photoelectron spectroscopy (XPS, ESCALAB 250Xi, Thermo Fisher, Waltham, MA, USA) in the range of 0 eV to 1350 eV, with a binding energy resolution of 0.1 eV.

### 2.4. Electrochemical Measurements

Electrochemical characterization of samples by using a three-electrode system on the same electrochemical workstation (CHI660E, Shanghai Chenhua, China), and the cycling stability was studied making use of a LAND battery system (CT2001A, Wuhan). 0.5 M Na_2_SO_4_ neutral solution was chosen as the aqueous electrolyte. The prepared LMO/MnO electrode was taken as the working electrode, the platinum electrode and a saturated calomel electrode (SCE) acted as the counter electrode and the reference electrode, respectively. Cyclic voltammetry (CV) was performed at various scan rates, ranging from 5 mV·s^−1^ to 100 mV·s^−1^, within a potential range of 0 to 1V. Galvanostatic charge-discharge (GCD) was tested at different current densities (0.5, 1, 2, 3, 4 A·g^−1^) to analyze charge/discharge times in a potential window from 0 to 1 V. Electrochemical impedance spectroscopy (EIS) test was performed with a frequency range of 10^−2^ Hz to 10^5^ Hz. In addition, a symmetric supercapacitor cell was also fabricated, separating two LMO/MnO electrodes about 0.5 cm, and the electrochemical performance was characterized as before.

The mass specific capacitance of the single electrode can be calculated from CV or GCD measurement on the basis of Equations (1) and (2), respectively [30,31].

(1) $$C_{\mathrm{single}}\;=\;\int_{V1}^{V2}idV/\left(2m\;\times \;s\;\times \;\Delta V\right)$$

(2) $$C_{\mathrm{single}}\;=\;(I\;\times \;\Delta t)/\left(m\;\times \;\Delta V\right)$$

where *i* (or *I*) is the measured current (A), *m* is the mass of the active materials, *s* is the potential scan rate (V·s^−1^), Δ*V* is the potential range, and ∆*t* is the discharge time (s).

The mass specific capacitance of symmetric supercapacitor cell can be calculated according to equation [32]:

(3) C = I/[m(dU/dt)]

Here C ($F\cdot g^{-1}$) represents mass specific capacitance, m (Kg) is the total mass of active material, dU/dt is the slope of the entire discharge curve form GCD.

Accordingly, the mass energy density *E* (Wh·kg^−1^) and power density *P* (W·kg^−1^) of supercapacitor cell can be expressed according to Equations (4) and (5) [32]:

(4) $$E=\int_{t1}^{t2}IVdt=0.5C\left(V1+V2\right)\left(V2-V1\right)\;$$

(5) P=E/Δt

where V1 and V2 are respectively the charging end voltage and discharge end voltage from GCD curve, (V2−V1) should be the specific voltage window of the capacitive behavior of the supercapacitor device, Δt is the discharge time (s).

## 3. Results and Discussion

### 3.1. Phase Structure and Morphology

**Figure 1:**
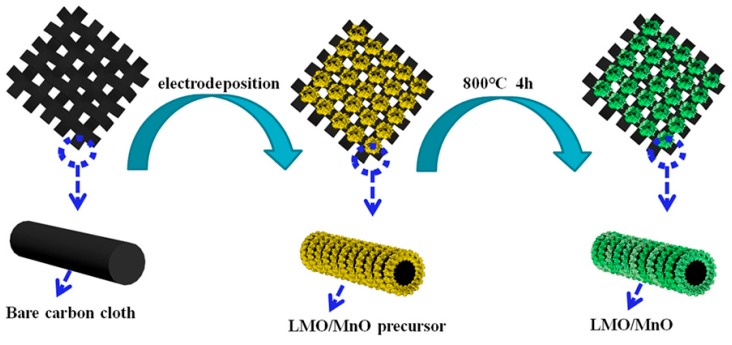
Schematic representation for the preparation of LMO/MnO electrode.

Electrochemical synthesis is a convenient and inexpensive way for the preparation of perovskite oxide coating. However, for La-based perovskites La*B*O_3_ preparation, the greatest weakness lies in the nonparticipation of La ions in any redox process reaction during the reaction. Thus, bath solution with a high La/*B* ratio is necessary for the steady electrodeposition of La*B*O_3_ [29]. In addition, *B*O*x* can strengthen the adhesion between the substrate and the active layer. *B*O*x* penetrates into the carbon fiber along the grain boundary and acts as a “pinning” to enhance the compatibility, which is beneficial to improve the life and stability of the electrode [12,28]. Therefore, for the LMO/MnO composite material obtained in the present work by cathodic deposition and annealing, the presence of MnO can increase the adhesion of LMO to the substrate. A schematic diagram showing the fabrication of LMO/MnO on a flexible carbon cloth is shown in Figure 1. A precursor film composed of mixed metal hydroxide is firstly obtained on the carbon cloth after electrodeposition. Then the precursor transforms into LMO/MnO composite material after a thermal treatment. The electronic photograph in Figure S1 shows the excellent flexibility of the obtained carbon cloth-supported LMO/MnO electrode.

**Figure 2:**
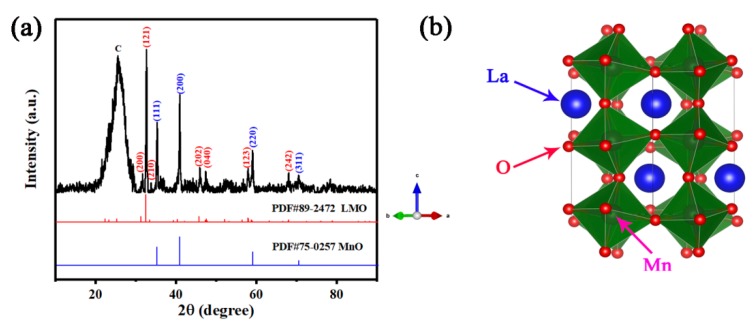
(**a**) XRD pattern of LMO/MnO electrode prepared from bath with 2 M La(NO_3_)_3_ and 0.1 M Mn(NO_3_)_2_; (**b**) the crystal structure of LMO.

Figure 2a shows the XRD patterns of carbon cloth-supported LMO/MnO electrode, where the phase composition and structures are confirmed. The broad peak around 25° corresponds to the carbon cloth, coincides with some previous reports which contain active materials grown on carbon cloth [33,34]. From XRD patterns, LMO is indexed to be orthorhombic with the space group of Pnma (JCPDS 89-2472), and MnO exhibits a cubic structure with $Fm\bar{3}m$ space group (JCPDS 75-0257). The corresponding crystal planes are indexed in Figure 2a. According to the space group, the crystal structure of the orthorhombic perovskite LMO is drawn by VESTA software as shown in Figure 2b. The distortion of [MnO_6_] octahedron along the c-axis is attributed to the Jahn-Teller effect of Mn^3+^ (3d^4^) [35,36,37].

**Figure 3:**
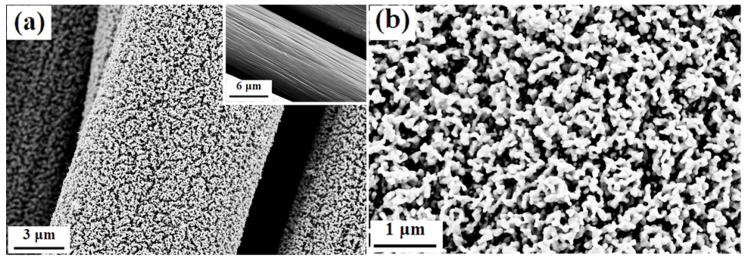
(**a**) SEM images of deposited LMO/MnO electrode prepared from bath with 2 M La(NO_3_)_3_ and 0.1 M Mn(NO_3_)_2_; inset shows bare carbon cloth fiber; (**b**) morphology in high magnification.

Deposit morphology also plays an indispensable role in optimizing the electrochemical properties of materials. The uniformity and thickness of LMO/MnO nano-arrays can be controlled by adjusting the La/Mn ratio of the electrodeposition solution. Figure 3 shows the deposit morphology of LMO/MnO electrode with the La/Mn ratio of 20:1 in bath solution. It can be seen that short rod-like interwoven nanoarrays are uniformly deposited on the bare carbon cloth fiber with the average deposition thickness of about 150 nm. The loose structure with increased specific surface area surely will provide more effective ion transfer channels and facilitate the electrochemical dynamic process, thereby improving the specific capacitance. Meanwhile, SEM images of the electrodeposited LMO/MnO electrode in bath solution with different La/Mn ratios (200:1 and 400:1) are shown in Figure S2. The nonuniform morphology may lead to the interruption of ions transport and weak adhesion to carbon cloth, adversely affecting the electrochemical properties, which will be discussed later. Considering the optimum morphology is obtained with the La/Mn ratio of 20:1, the following discussions are mainly focused on the optimum LMO/MnO electrodes.

**Figure 4:**
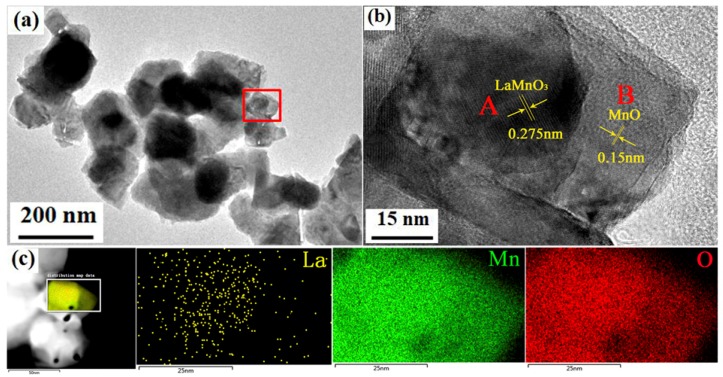
(**a**) Bright-field image of LMO/MnO nanoparticles prepared from bath with 2 M La(NO_3_)_3_ and 0.1 M Mn(NO_3_)_2_; (**b**) HRTEM image of the marked area; (**c**) STEM-EDS mappings of the marked area for different elements of La, Mn and O.

TEM analysis in Figure 4 shows more detailed information both in morphology and structure. The bright field image in Figure 4a shows the dissolved LaMnO_3_ and MnO particles are nanosized, consistent with the morphology observed in Figure 3b. In order to determine the distribution of LaMnO_3_ and MnO, detailed analysis has been conducted in the selected area marked by the red rectangle. The magnified HRTEM image of the designated area is shown in Figure 4b, the contrast between region A and region B is apparent. The lattice spacing of the region A is about 0.275 nm, which corresponds to the (121) plane of the orthorhombic LMO [38]. While the lattice spacing of 0.15 nm in region B is associated with the (220) interplanar spacing of MnO [39]. The structural analysis from TEM observations is in good agreement with XRD analysis. The results of scanning TEM microscopy (STEM) and EDS elemental analysis (Figure 4c) show a good spatial distribution of La, Mn, and O elements throughout the designated area. It can be found that La element mainly distributes in region A, but is barely observed in region B. Slight errors may exist during the mapping because prolonged exposure to the electron beam may result in slight sample drift. A linear component analysis through the designated area has also been conducted (Figure S3), leading to the same conclusion that region A is LMO and region B represents MnO. It can also be speculated that LaMnO_3_ and MnO distribute randomly during electrodeposition.

**Figure 5:**
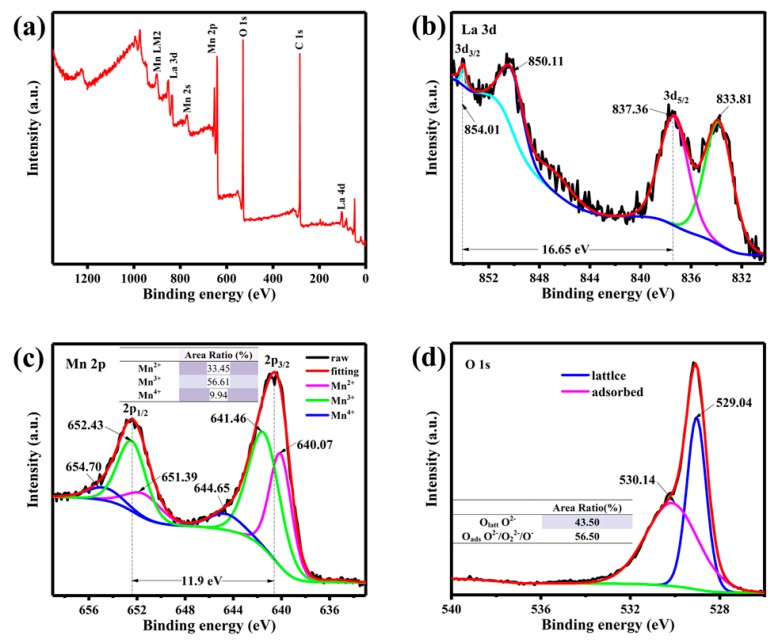
(**a**) Full XPS spectra; (**b**) La 3d; (**c**) Mn 2p; and (**d**) O1s peaks in deconvoluted XPS spectra of LMO/MnO electrode prepared from bath with 2 M La(NO_3_)_3_ and 0.1 M Mn(NO_3_)_2_.

The results of XPS analysis presented in Figure 5 show the chemical valence and elemental composition of LMO/MnO electrode. Figure 5a shows a measurement scan of C(1s), La(3d), Mn(2p), O(1s) in the LMO/MnO electrode in the range of 0 eV to 1350 eV, indicating that no impurities are present. As shown in Figure 5b, the La 3d spectrum shows two spin-orbital components of La 3d_5/2_ (833.81eV and 837.36 eV) and La 3d_3/2_ (850.11 eV and 854.01 eV) in the scan range of 830 eV to 855 eV. The observation is similar to those of La-based perovskite materials reported previously [19], and indicates the existence of La^3+^ ions [40]. The Mn 2p spectrum (Figure 5c) shows two peaks at 640.53 eV and 652.43 eV with a binding energy separation of 11.9 eV, corresponding to Mn 2p_3/2_ and Mn 2p_1/2_, respectively [20]. The two peaks of the Mn 2p spectrum within the scanning range of 633 eV to 659 eV are fitted with Mn ions of three valence states: Mn^2+^, Mn^3+^, and Mn^4+^. Peaks which appear at 641.2 eV and 652.3 eV are related to Mn^2+^, peaks at 642.4 eV and 654.0 eV correspond to Mn^3+^, while peaks at 644.0 eV and 655.5 eV represent Mn^4+^. The relative concentrations for Mn^2+^, Mn^3+^, and Mn^4+^ are shown in the inset of Figure 5c. It has been proven that the coexistence of Mn^4+^ and Mn^3+^ can improve the conductivity of LMO, thus an optimized electrochemical performance can be expected. The O1s spectrum has two peaks in the range of 526 eV to 540 eV (Figure 5d), which are binding energies of 529.04 eV and 530.14 eV, respectively, corresponding to lattice oxygen O_latt_ (O^2−^) and oxygen absorption O_ads_ (O^−^, O_2_^−^ or O_2_^2−^), respectively [41,42]. A high O_ads_ concentration of 56.50% is obtained in the present work, indicating the stronger adsorption capacity of OH^−^, which accelerates the surface redox reaction kinetics and improves the electrochemical performance.

### 3.2. Electrochemical Performance

**Figure 6:**
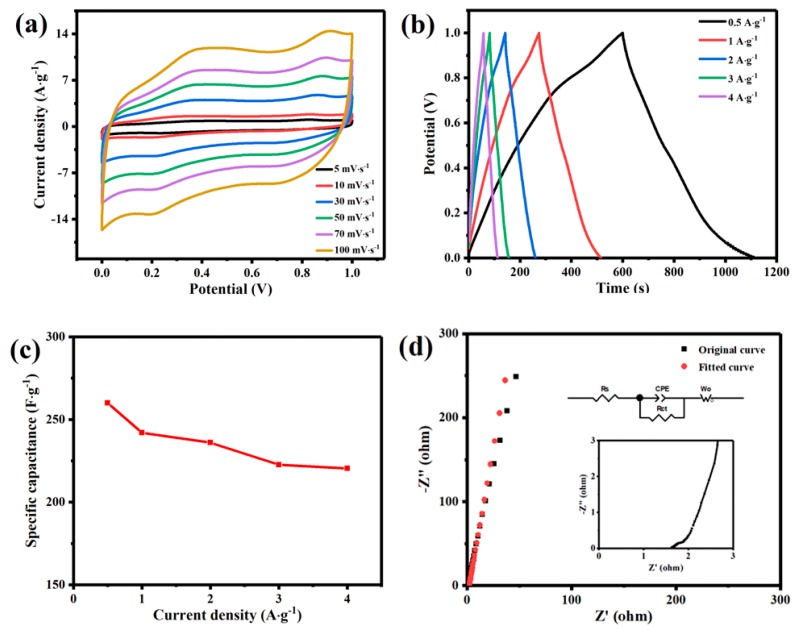
Electrochemical behavior of LMO/MnO electrode prepared from bath with 2 M La(NO_3_)_3_ and 0.1 M Mn(NO_3_)_2_ in 0.5 M Na_2_SO_4_ solution: (**a**) CV curves at different scan rates; (**b**) GCD curves at different current densities; (**c**) dependence of specific capacitance vs. current density; and (**d**) Nyquist plot at open circuit potential; the inset in Figure 6d shows the proposed equivalent circuit with components discussed in the text.

In order to reflect the real electrochemical performance of LMO/MnO composite active materials, the electrochemical behavior of the bare carbon cloth in 0.5 M Na_2_SO_4_ solution has also been investigated. Figure S4 shows CV curves of the carbon cloth supported LMO/MnO electrode and bare carbon cloth at 50 mV·s^−1^. It is obvious that the bare carbon cloth shows a relatively low double-layer capacitance, and its contribution to the LMO/MnO electrode is negligible, thus the electrochemical performance of the active material can be truly reflected. Figure 6a shows CV curves of the deposited LMO/MnO electrode in 0.5 M Na_2_SO_4_ aqueous electrolyte at different scan rates from 5 mV·s^−1^ to 100 mV·s^−1^ with a potential range of 0 to 1 V. The CV curves in this Figure show two redox couples with the oxidation peaks at 0.36 V and 0.91 V and the reduction peaks at 0.2 V and 0.78 V. As XPS analysis demonstrates three different oxidation states of Mn, these peaks correspond to the transformations between different oxidation states of Mn ions. Peaks at 0.36 V and 0.2 V correspond to the oxidation and reduction of Mn^2+^ and Mn^3+^, while peaks at 0.91 V and 0.78 V represent the redox of Mn^3+^ and Mn^4+^. According to the anion-intercalation mechanism, the redox reaction of perovskite LMO during the charge/discharge process can be expressed as following [10,15,43]:

(6) $$\mathrm{La}\left[{\mathrm{Mn}}_{2\delta }^{2+};{\mathrm{Mn}}_{1-2\delta }^{3+}\right]O_{3-\delta }+2{\delta \mathrm{OH}}^{-}↔{\mathrm{LaMn}}^{3+}O_{3}+2{\delta e}^{-}+{\delta H}_{2}O$$

(7) $${\mathrm{LaMn}}^{3+}O_{3}+2{\delta \mathrm{OH}}^{-}↔\mathrm{La}\left[{\mathrm{Mn}}_{2\delta }^{4+};{\mathrm{Mn}}_{1-2\delta }^{3+}\right]O_{3+\delta }+2{\delta e}^{-}+{\delta H}_{2}O$$

The perovskite-type LMO stores energy through the oxygen-vacancy tailored redox pseudocapacitance, and the ion diffusion along the oxygen octahedral edges confirms the high diffusion rate and full utilization of the internal structure [44]. In addition, MnO has also been reported as a negative electrode material with good properties. Studies have shown that MnO nanoparticles uniformly distributed in the carbon nanoshells can greatly improve the transfer kinetics of charge carriers and structural stability during discharge/charge cycle [45]. The redox reaction of MnO in charging and discharging process can be expressed as following [46]:

(8) $${\mathrm{Mn}}^{2+}↔{\mathrm{Mn}}^{3+}+e^{-}$$

(9) $${\mathrm{Mn}}^{3+}↔{\mathrm{Mn}}^{4+}+e^{-}$$

The specific capacitance in the present work is attributed to the cooperative effects of LMO and MnO.

The charge and discharge curve (GCD) is also an important parameter to measure the practicality of supercapacitors. Figure 6b shows GCD curves of LMO/MnO electrode at different current densities, all curves are basically triangularly symmetrical, which ensure high rate performance of LMO/MnO electrode. As the current density increases, the specific capacitance shows a downward trend, due to the increase of internal resistance. The obtained capacitance for single electrode with the variation of current density are shown in Figure 6c. According to the discharge curves at current densities of 0.5, 1, 2, 3, and 4 A·g^−1^, the corresponding capacitances are 260, 242, 236, 222.6, and 220.4 F·g^−1^, respectively. Comparatively, the GCD curves of LMO/MnO materials in bath solution with different La/Mn ratios (200:1 and 400:1) are shown in Figure S5. The specific capacitances are calculated as 76 F·g^−1^ (0.5 A·g^−1^) and 134 F·g^−1^ (0.5 A·g^−1^), respectively. It can be concluded that the uniform deposition morphology is favorable for the optimization of specific capacitance. A typical Nyquist plot of the LMO/MnO electrode in 0.5 M Na_2_SO_4_ solution is shown in Figure 6d. There are three parts make up of this EIS diagram (see the inset in Figure 6d). At higher frequency, the x-intercept with the initial curve is called the effective series resistance (R_s_) [47,48]. It is commonly used as the bulk resistance of electrochemical systems, which includes electrolyte and internal resistance of the electrode. The second part is the semicircle of the intermediate frequency region, and its diameter corresponds to the charge transfer resistance (R_ct_) of the electrode/electrolyte interface [49,50]. The third part of the EIS diagram is the linear portion of the low frequency region, representing the proton diffusion in the active material, as a Warburg impedance W_o_ in the equivalent circuit [51]. Impedance spectroscopy has been simulated by appropriate equivalent circuit diagram. The ohmic resistance (R_s_) is calculated as 1.61 Ω, suggesting ion transport between LMO/MnO electrode and electrolyte is less hindered. R_ct_ is calculated as 0.10 Ω, indicating that the charge transfer resistance is very low at the open-circuit potential, the electron activity in the electrode material is high and the conductivity of the material is remarkable. In addition, ion diffusion impedance Wo (Warburg impedance) characterized in the low frequency region is 1.26 Ω, suggesting good behavior of the electrode. The determined value of the constant phase element CPE (instead of double layer capacitance) is 3.02 µF, showing good capacitance behavior. The analysis of Nyquist diagram indicates the low resistance of LMO/MnO electrode, confirming the excellent electrochemical capacitance behavior.

**Figure 7:**
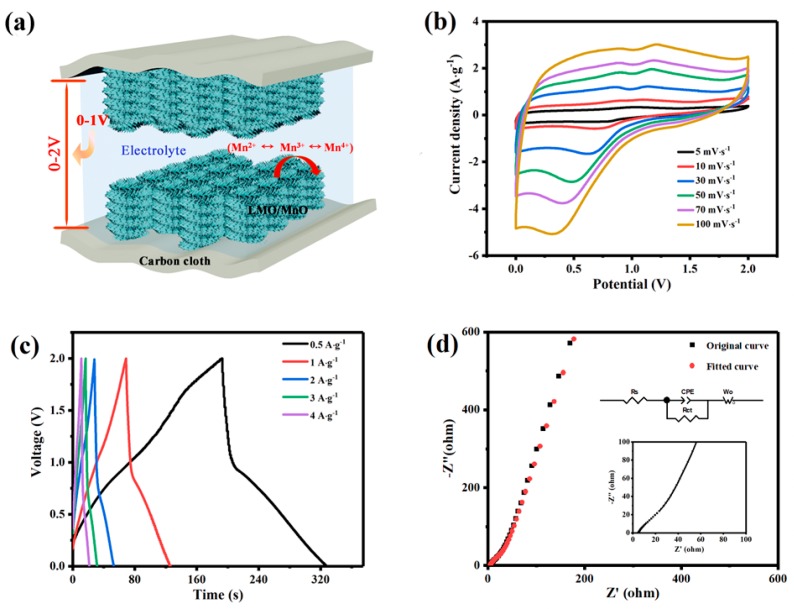
(**a**) Schematic diagram of LMO/MnO symmetrical supercapacitor cell in 0.5 M Na_2_SO_4_; (**b**) CV curves at different scan rates; (**c**) GCD curves at different current densities; and (**d**) EIS Nyquist plot of symmetrical cell. The electrodes were prepared from bath with 2 M La(NO_3_)_3_ and 0.1 M Mn(NO_3_)_2_.

**Figure 8:**
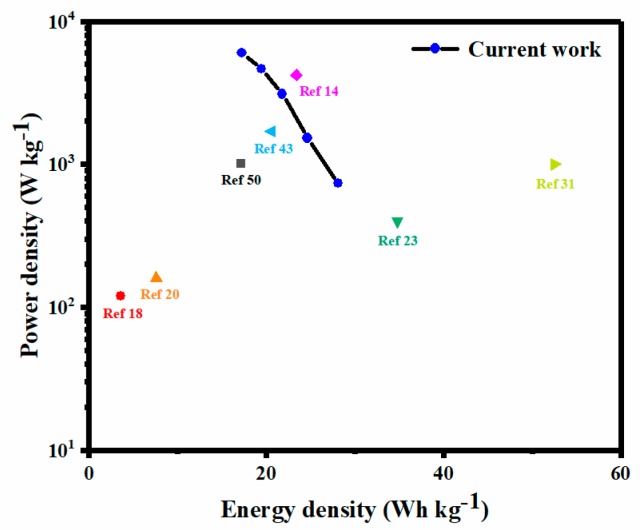
Ragone plots of some recently reported supercapacitors based on perovskite oxides compared with the present data.

To further study the application of LMO/MnO electrode in supercapacitors, a symmetric supercapacitor has been fabricated in 0.5 M Na_2_SO_4_ solution, as shown in Figure 7a. For the symmetric supercapacitor, the operation voltage window (V) doubles to 2 V, larger than other supercapacitors reported previously [20,23], and a high energy density (E) is expected, based on the equation $E=\int_{t1}^{t2}IVdt=0.5C\left(V1+V2\right)\left(V2-V1\right)$. The deviation of the CV curves from the ideal shape (Figure 7b) is due to the insufficient time to intercalate/deinsert the active species at each site [31]. The increase of solution resistance results in an obvious voltage drop in charge–discharge curves (Figure 7c). The specific capacitances of the LMO/MnO symmetric supercapacitor obtained at 0.5, 1, 2, 3, and 4 A·g^−1^ are 50.66, 44.43, 39.32, 35.09, and 31.10 F·g^−1^, respectively. The maximum energy density of 28.15 Wh·kg^−1^ is obtained when the power density is 745 W·kg^−1^, and maintains in 17.28 Wh·kg^−1^, even when the power density is 6100 W·kg^−1^. The Nyquist plot in Figure 7d exhibits an increased resistance of LMO/MnO symmetric supercapacitor, which may originate from the contact impedance between electrodes. Ragone plots of several recently reported supercapacitors based on perovskite oxides compared with the present data are shown in Figure 8. The performance of the carbon cloth-supported LMO/MnO supercapacitor in the present work is comparability, demonstrating the great potential toward practical application of supercapacitors.

**Figure 9:**
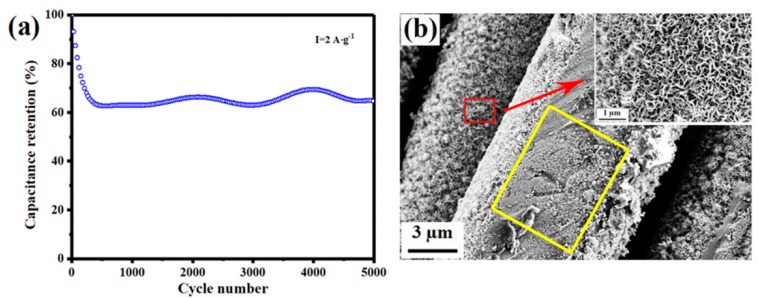
(**a**) Cycling performance at a current density of 2 A·g^−1^; (**b**) SEM image of LMO/MnO electrode after 5000 cycles.

The long-term cycling stability is also a key parameter to characterize the electrochemical performance of supercapacitors. The cycling stability of LMO/MnO symmetric supercapacitor with the current density of 2 A·g^−1^ is investigated and shown in Figure 9a. Interestingly, the specific capacitance drops to 65% retention after the first 500 cycles, and remains stable until 5000 cycles. For perovskite oxides, an important factor that weakens the cyclic stability is the element leaching effect into electrolyte, which may cause the damage of perovskite structure [18,52]. The EDS analysis before and after cycles in Figure S6 shows the significant reduction of La element after 5000 cycles, indicating the element leaching phenomenon in the present work. On the other hand, the morphology of active material also has impact on the cycling stability. Figure 9b shows the SEM image of the electrode morphology after 5000 cycles. It can be obviously found that LMO/MnO active materials partially flake from carbon cloth after cycles, as the flaking area is marked with yellow square. The decrease of active materials and the effective contact of the electrolyte with the active material may lead to the drop of specific capacitance at first 500 cycles. However, the remaining active materials adhere firmly, and the short rod-like interwoven morphology (Figure 3) changes to the hierarchical flower-like morphology. Various materials with the similar flower-like morphology have been reported, showing excellent electrochemical performance with superior cycling stability [53,54,55]. Thus, the newly formed flower-like morphology inhibits the deterioration of the specific capacitance. In the process of repeated charge and discharge, the original diffusion channels are destructed and new channels are reconstructed, leading to the small fluctuations of the specific capacitance. After 5000 cycles, 65% of the original capacitance is retained. The enhancement of the adhesion strength between substrate and active materials may improve the cycling stability of the present electrode.

## 4. Conclusions

In this work, carbon cloth supported perovskite-type LaMnO_3_/MnO (LMO/MnO) electrode has been prepared via a simple one-step electrodeposition method. The phase structure, deposition morphology, and the corresponding electrochemical properties of LMO/MnO electrode have been investigated. LMO/MnO electrode exhibits a high specific capacitance (260 F·g^−1^ at 0.5 A·g^−1^) and excellent rate performance in an aqueous electrolyte. The specific capacitance is attributed to the cooperative effects of LMO and MnO, as well as the uniform nano-array morphology. In addition, a wide operation voltage window of 2 V is obtained in the LMO/MnO symmetric supercapacitor, which shows a high energy density of 28.15 Wh·kg^−1^ at a power density of 745 W·kg^−1^. The specific capacitance drops to 65% retention after the first 500 cycles because of the element leaching effect and partial flaking of LMO/MnO, yet remains stable until 5000 cycles due to the formation of the flower-like morphology. The present work provides a new structural form of perovskite oxides as electrodes for supercapacitors, and demonstrates the great potential of La-based perovskites in the field of rapid energy storage.

## Supplementary Materials

The following are available online at https://www.mdpi.com/2079-4991/9/12/1676/s1, Figure S1: Electronic photograph of LMO/MnO electrode material prepared from bath with 2 M La(NO_3_)_3_ and 0.1 M Mn(NO_3_)_2_; Figure S2: SEM images of LMO/MnO electrode with different La/Mn ratios (a) La/Mn = 200:1 (b) La/Mn = 400:1; Figure S3: Relative elements content in STEM-EDS line scanning across LMO/MnO electrode prepared from bath with 2 M La(NO_3_)_3_ and 0.1 M Mn(NO_3_)_2_; Figure S4: CV curves of carbon cloth and LMO/MnO electrode material (prepared from bath with 2 M La(NO_3_)_3_ and 0.1 M Mn(NO_3_)_2_) at 50 mV·s^−^^1^; Figure S5: GCD curves at different La/Mn ratios of LMO/MnO electrode (a) La/Mn = 200:1 (b) La/Mn = 400:1; Figure S6: The EDS analysis before and after the cycles.
