# Supplementary material for: Flexible Supercapacitor Electrodes Based on Carbon Cloth-Supported LaMnO3/MnO Nano-Arrays by One-Step Electrodeposition
Source: Nanomaterials (Basel). 2019 Nov 24;9(12):1676. doi: 10.3390/nano9121676 (PMC6956280; doi:10.3390/nano9121676)
Supplement: Supplementary file 1 [file nanomaterials-09-01676-s001.pdf]

Supplementary Information

Flexible Supercapacitor Electrodes Based on Carbon Cloth-Supported $LaMnO_3$/MnO Nano-Arrays by One-Step Electrodeposition

Pianpian Ma[1,2,*], Na Lei [1], Bo Yu [1], Yongkun Liu [1], Guohua Jiang [1,2,*], Jianming Dai [1], Shuhong Li [1] and Qiuling Lu [1]

[1] School of Materials Science and Engineering, Zhejiang Sci-Tech University, Hangzhou 310018, China; A17857121453@126.com (N.L.); eric_yubo@126.com (B.Y.); 17826854303@163.com (Y.K.L.); d2239961778@gmail.com (J.M.D.); shuhongli2018@126.com (S.H.L.); lqllql123lql@163.com (Q.L.L.)

[2] Key Laboratory of ATMMT Ministry of Education & National Engineering Laboratory for Textile Fiber Materials and Processing Technology & Institute of Smart Fiber Materials, Zhejiang Sci-Tech University, Hangzhou 310018, China

* Correspondence: pianpianma@zstu.edu.cn (P.P.M.), ghjiang_cn@zstu.edu.cn (G.H.J.)

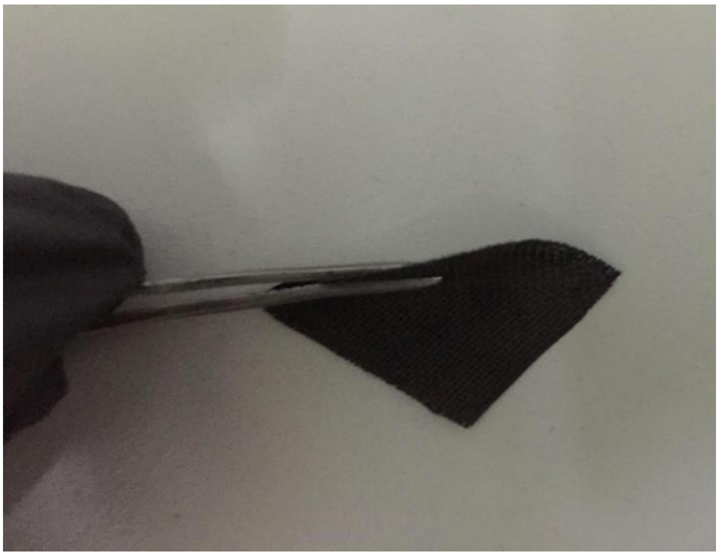

Figure S1. Electronic photograph of LMO/MnO electrode material prepared from bath with 2 M $La(NO_3)_3$ and 0.1 M $Mn(NO_3)_2$.

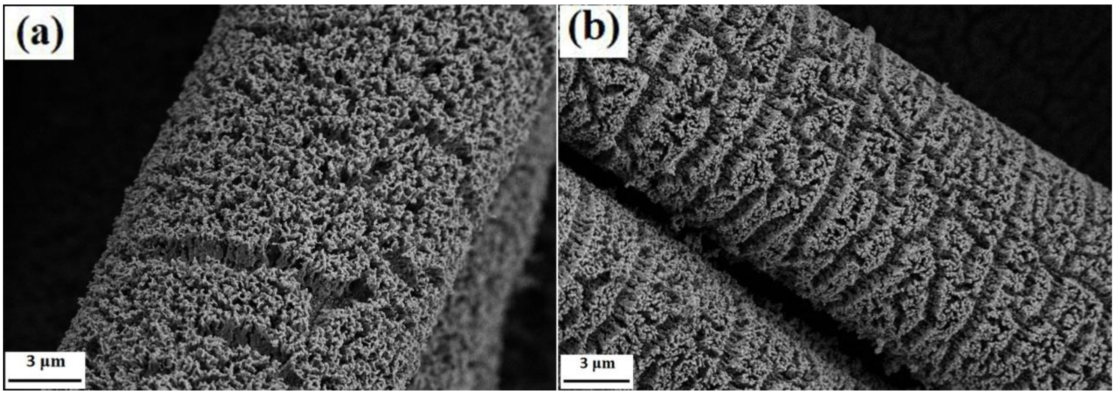

Figure S2. SEM images of LMO/MnO electrode with different La/Mn ratios (**a**) La/Mn = 200:1 (**b**) La/Mn = 400:1.

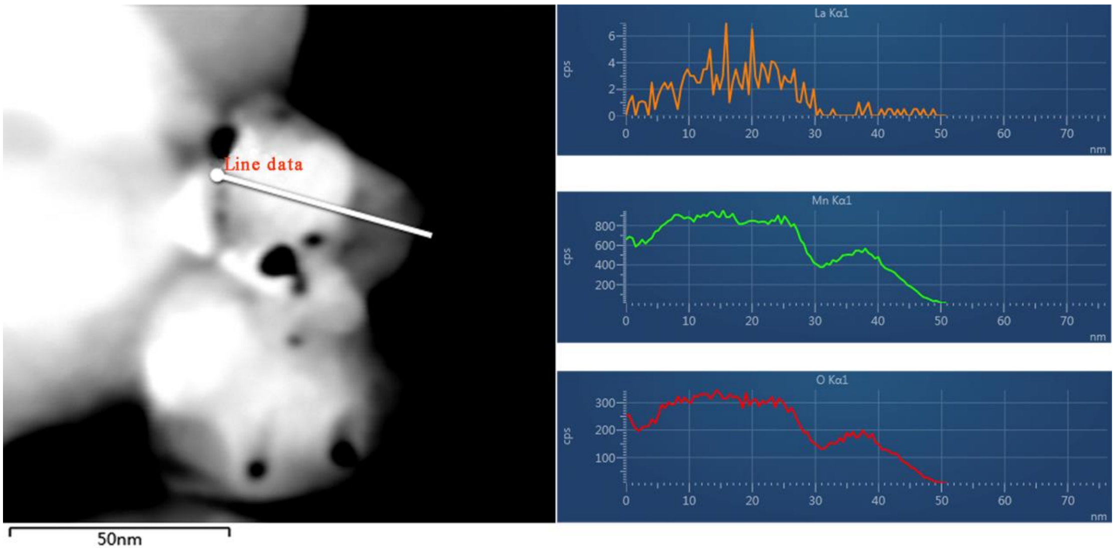

Figure S3. Relative elements content in STEM-EDS line scanning across LMO/MnO electrode prepared from bath with 2 M $La(NO_3)_3$ and 0.1 M $Mn(NO_3)_2$.

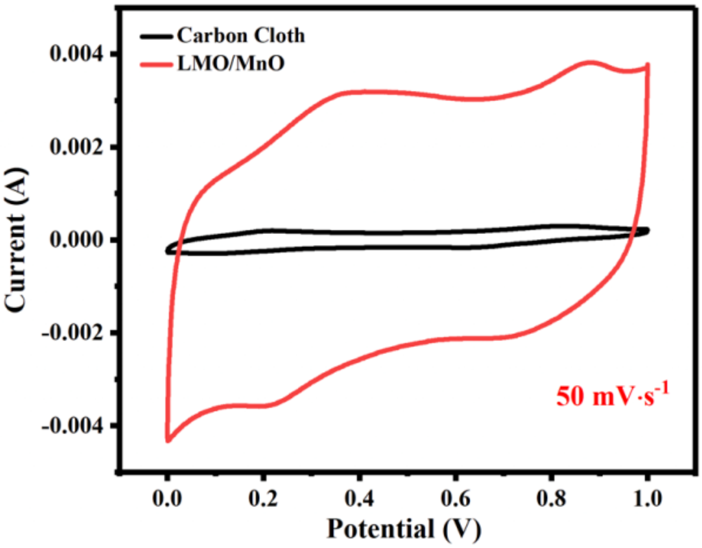

Figure S4. CV curves of carbon cloth and LMO/MnO electrode material (prepared from bath with 2 M $La(NO_3)_3$ and 0.1 M $Mn(NO_3)_2$) at 50 $mV{\cdot}s^{-1}$.

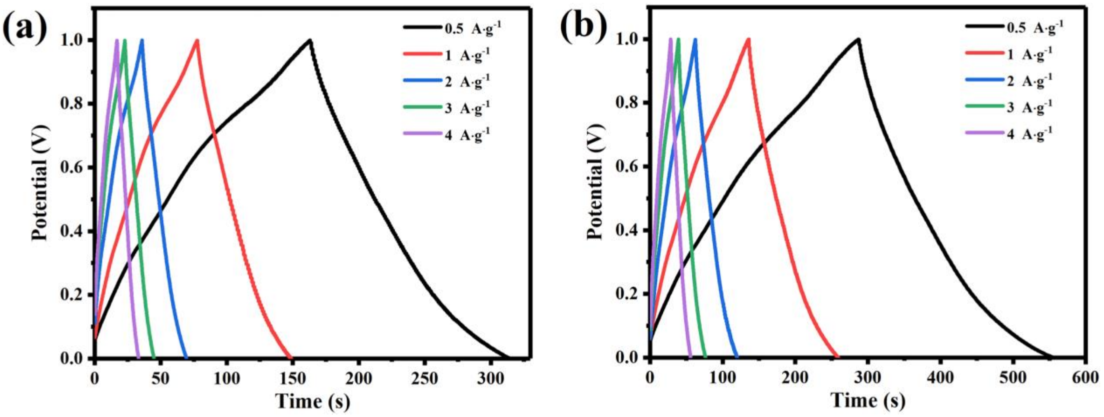

Figure S5. GCD curves at different La/Mn ratios of LMO/MnO electrode (**a**) La/Mn = 200:1 (**b**) La/Mn = 400:1.

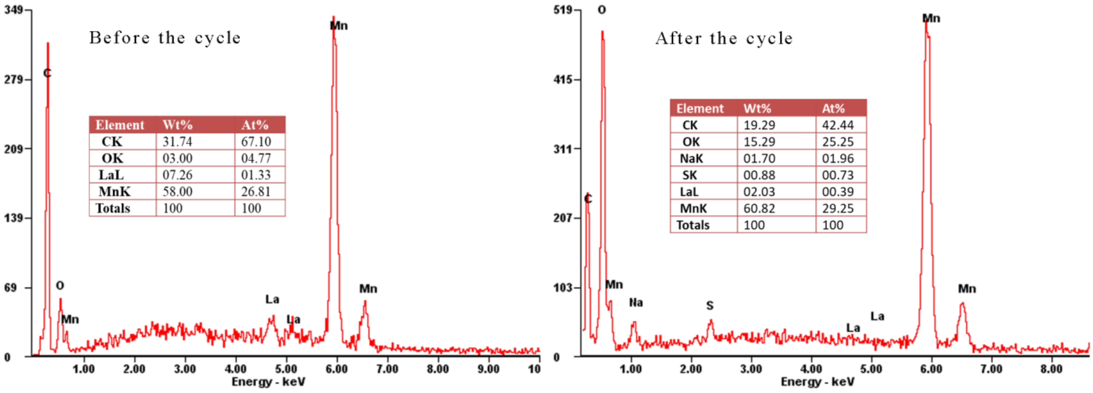

Figure S6. The EDS analysis before and after the cycles.
